# Coronavirus Disease 2019: Exploring Media Portrayals of Public Sentiment on Funerals Using Linguistic Dimensions

**DOI:** 10.3389/fpsyg.2021.626638

**Published:** 2021-02-18

**Authors:** Sweta Saraff, Tushar Singh, Ramakrishna Biswal

**Affiliations:** ^1^Amity Institute of Psychology and Allied Sciences, Amity University, Kolkata, India; ^2^Department of Psychology, Banaras Hindu University, Varanasi, India; ^3^Department of Humanities and Social Sciences, National Institute of Technology Rourkela, Sundargarh, India

**Keywords:** sentiment analysis, COVID-19, LIWC, SEANCE, social cognition, affect, fear and disgust

## Abstract

Funerals are a reflective practice to bid farewell to the departed soul. Different religions, cultural traditions, rituals, and social beliefs guide how funeral practices take place. Family and friends gather together to support each other in times of grief. However, during the coronavirus pandemic, the way funerals are taking place is affected by the country's rules and region to avoid the spread of infection. The present study explores the media portrayal of public sentiments over funerals. In particular, the present study tried to identify linguistic dimensions associated with lexical components of social processes, affective processes, fear, and disgust. An exhaustive search of newspaper coverage of funeral and related articles was made for a specific corona period. After an initial screening for the details and language used, a total of 46 newspaper articles on funerals were finalized for the analysis. Linguistic Inquiry and Word Count (LIWC) software was used to determine the association between linguistic dimensions of function words and words related to social and affective processes, as presented in the newspaper articles. Sentiment Analysis and Cognition Engine (SEANCE) was applied for the analysis of sentiment, social cognition, and social order. Bayesian correlation analysis and regression revealed positive and significant associations between function words and affective processes, between pronouns and social processes, and between negative adjectives and psychological processes of fear and disgust. Also, significant negative associations were found between polarity nouns and psychological processes of fear and disgust and between polarity verbs and psychological processes of fear and disgust. Bayes factor 10 provides strong evidence in favor of the study hypotheses. The media is influenced by the prevailing sentiments in society and reflects their perception of the current social order and beliefs. The findings provide a glimpse into the prevailing sentiment of society through the lens of media coverage. These understandings are expected to enhance our observations of how people express their feelings over the loss of their loved ones and help mental health professionals develop their therapeutic protocols to treat the coronavirus disease 2019 (COVID-19)-affected population.

## Introduction

The coronavirus pandemic changed our daily lives, work behavior, social gathering, and customary funeral traditions. Bidding a ritualistic farewell with prayers for peace and forgiveness is a convention followed since time immemorial, irrespective of religions. This period overwhelms the entire family, relatives, and friends with grief. However, during the coronavirus disease 2019 (COVID-19) pandemic, this age-old practice of offering a farewell to deceased relatives is also affected. Due to the pandemic and the fear of infections, funerals and burials are either postponed or held remotely in most parts of the world. The family members are not present in these rituals and thus are denied opportunities to offer a final goodbye (Bhanot et al., 2020). Under these circumstances, families are anguished, especially when they cannot share their loss with other families/society (Wallace et al., 2020). Literature suggests that the inability to perform the funeral rituals and bid farewell to deceased family members/relatives due to limited exposure or fear of infection results in the feeling of self-blame, grief, and anger (Wallace et al., 2020). The literature related to previous pandemics also suggests similar outcomes. The Commission to Investigate the Introduction and Spread of Severe Acute Respiratory Syndrome (SARS), and Campbell (2006) on the outbreak of severe acute respiratory syndrome-related coronavirus in 2002–2004 noted in its final report that “those left behind had no opportunity to confront the reality of death and to honor the life of the deceased” (p. 943), “with the proviso that funeral rites must obviously carry lower priority than the need to contain the virulent public health threat” (p. 942).

These experiences of grief, self-blame, and anger are heightened as the stories covered by the media and content shared on social media platforms use languages that present an emotional distance to who will contract and/or die from COVID-19 infection (Wallace et al., 2020). During the pandemic, the sensation-seeking and fear-mongering behavior of the media impacted families' sentiments that will be remembered for years to come. It created a wave of emotions through print and television news, and social media shares that people were afraid of burying their deceased family members. The fear of infection again reminded us that our survival is important.

The present study was conducted to highlight how, through language frameworks and emotional contents, the media influences social order throughout the world, extending to funeral practices.

## Sentiment Analysis

Language shapes our choices, thinking style, and decision making and also differentiates humans from other primates. Dewey (1910) theorized language as a channel of thinking or vehicle for thought, and the philosophy of Freud (1953) encompasses emotions and motives intricately woven around the dreams of a person communicated through language to the listener. McClelland et al. (1953) used language categories such as achievement, affiliation, and power to assess individual needs (Lasswell et al., 1952) by clustering words such as triumph, conquest, prevail, and accomplish victory. Wilhelm von Humboldt (1767–1835) penned, “Language is the outward manifestation of the spirit of people: their language is their spirit, and their spirit is their language; it is difficult to imagine any two things more identical” (in Salzmann, 2004:42). Through diverse research and publications on the human expression of written language, Pennebaker and King (1999), Pennebaker (2004), and Pennebaker et al. (2014, 2015) showed that computerized methods analyze semantics and syntax in language formats.

The prevalence of enormous and unmanageable data along with the need for probabilistic analytical tools like Bayes resulted in a significant rise in the application of machine-based language tools. According to Taraban et al. (2018), “The reliance on probabilistic representations of linguistic features forms a common ground in human and machine-based language processing.” NLP or natural language processing refers to artificial intelligence (AI) to process and analyze written or spoken language (Taraban et al., 2018). Word2Vec is a neural network that transforms text inputs into vectors (Mikolov et al., 2013; Pal et al., 2020). Rationality vs. emotional style of thinking can be analyzed through NLP (Cambria et al., 2010a; Akhtar et al., 2020). Emotion words such as glad, fear, blue, eagerness, excitement, agony, alarm, anguish, desire, disgust, and joy are categorized based on syntactic structure. Linguistic Inquiry and Word Count (LIWC) is a supervised learning machine-based tool that provides in-depth information on opinions, thinking style, affect, and cognitive processes. It can objectively quantify text messages, both syntactically and semantically. Sentiment Analysis and Cognition Engine (SEANCE) is another machine-based algorithmic application that analyses sentiments, social behavior, and cognitive processes. The present study was planned to understand the underlying association between the linguistic frame of newspaper articles and their subliminal impact, whether implied or not, on the public. This pandemic has seen the untimely demise of closed ones in many families; the reason is a lack of understanding about the nature of the virus and fear-mongering by governments and the media. Thus, we attempt to utilize two machine-based supervised learning tools to analyze the newspaper texts to comprehend the underlying psychological processes. The specific objectives of the study were (a) to understand the association between the human affective process and the use of function words in newspaper articles, (b) to comprehend the strength of an association between pronouns and social processes through text analysis of newspaper articles, and (c) to study the relationship between fear, disgust, polarity nouns, polarity verbs, and adjectives.

## Setting the Context

The potential impact on the news media reporting the travesty of coronavirus shook the world from late 2019 to date. The world as a whole was grappling with fears of an unknown virus, and almost everyone was in voluntary home lockdown starting on March 25, 2020, in India. Citizens were dependent on news media reports regarding the number of people who were dying each day due to the virus. The medical system was overwhelmed with an increasing number of COVID-19 patients. Everyone was concerned about the health of their close ones and relatives. The situation was bleak when people who got infected or had cold or fever were taken by the medical team appointed by the state departments. The families were quarantined too and separated from the sick member; sometimes, they did not know each other's whereabouts. People were overwhelmed with this news all over television, print media, and social media shares. It was difficult to accept that a family could not cremate their deceased family member, who may be a parent, a grandparent, kin, or spouse. This article is about 1–3% of families who could not cremate their loved ones and how print media across the world and India reported it.

## The Literature Gap and the Research Questions

This study seeks to understand the potential influence that the media may have upon its readers through the language used in their articles on affect (human emotions), fear, and death. It is evident from the related literature on text analysis of newspaper articles that they have a marked influence on perceptions and health-associated issues (Cambria, 2016). The media's portrayals of funerals via their communication style and language use, especially the weightage on each “part of speech” (POS), make it necessary to study their impact. However, text analysis of the media's portrayals of funerals through print has not been explored much, and work like this may give us a unique perspective (Jalilifar et al., 2014; Chaiuk and Dunaievska, 2020). With this work, we wish to seek answers to the following research questions: (a) does the written communication use more affect or emotion-laden words, or (b) does use of function words and adjectives stir deep emotions like fear and disgust? Using two machine-based learning tools, we analyzed text-based news coverage of funerals during the COVID-19 pandemic.

## Method

The sentiment analysis research used NLP techniques on 46 newspaper articles published on funeral practices during March–June 2020 and one article on South Korea in November 2019. There were stringent rules in India and worldwide related to burial customs across cultures during the COVID-19 lockdown. The text analysis of media content was done in this study to explore the semantic structure of the written content that can be gained through algorithmic applications (Taraban et al., 2019). The language pattern on sentiments and opinions (Pang and Lee, 2008) and the relationships between its context and interpretation (Wiebe and Mihalcea, 2006; Turney et al., 2011) have not been explored in news articles written on burials or funerals. Machine learning (ML) tools are used to extract linguistic annotations, like verbs, nouns, adverbs, and adjectives (Li et al., 2011; Hao et al., 2013). These linguistic features provide a source for sentiment analysis of the syntactic structure.

### Newspaper Selection

Popular newspapers that are also geographically diverse were selected from both India and other countries. The Indian newspapers that were included have both national and regional presence. The Indian papers are *Bangalore Mirror, Daily Mail, Devdiscourse, India Today, Indian Express, Mumbai Mirror, NDTV News, Pune Mirror, Scroll.in, The Hindu, The Print*, and *Times of India*. International articles were selected from Licas.news (Asian), BBC News (England, Brazil, Ghana, Italy, USA), MinnPost (USA), The Economic Times (USA), The Guardian (Australia, Ecuador, England, Ireland), *The Hindu* (South Korea), *The New York Times* (USA), Thomson Reuters (international news, South Africa), *Times of India* (China), *USA Today* (USA), and USNews (USA).

### Newspaper Article Selection

An exhaustive search of newspaper coverage of the funeral and related articles were made for a specific corona period, mostly from March to June 2020. After an initial screening for the details and language used, a total of 46 newspaper articles on funerals during the coronavirus pandemic were finalized for analysis. The keywords used for article selection were “funerals,” “burial,” “COVID 19,” “Coronavirus,” and “Newspaper Articles.” Google's search engine was used to extract the articles. After a detailed search, 46 articles were selected that covered different sentiments across varied cultures related to funeral or burial prohibitions imposed in most nations. In India, 19 articles were written on the situation across various cities during the particular strict lockdown phase (late March to early June), and 27 articles that reported on funeral practices during that period over the world were selected for the study.

### Linguistic Inquiry and Word Count

LIWC program (Linguistic Inquiry and Word Count: Pennebaker Conglomerates Inc, Austin, TX; LIWC, 2015), a computerized text analysis software that can be installed in the system's hard drive and data, can be processed without an internet connection. LIWC can process data in.txt format and can extract text data from a folder. LIWC dictionaries are available at http://dictionaries.liwc.net. It counts the word frequency and word stem to understand psychological processes of cognition, affect, and social elements (Francis and Pennebaker, 1992; Berry and Pennebaker, 1993; Pennebaker et al., 2015). Other than cognition, emotion, and personal concerns, the LIWC dictionary comprises nearly 6,400 words (Pennebaker et al., 2015), including positive and negative emotions, social, perceptual, and biological processes. The additional dictionaries recently included drives, time orientations, and the use of informal languages. It also has the advantage of composite categories for summary scores of analytic, clout, authentic, and tone, which are then converted to percentiles based on large samples. The linguistic dimensions include function words like pronouns, articles, adverbs, etc. Other grammatical features include verbs, adjectives, quantifiers, etc. LIWC has high content and construct validity (Francis and Pennebaker, 1992; Stirman and Pennebaker, 2001); inter-rater reliability also ranges between 86 and 100% relative to the dimension being assessed (Tausczik and Pennebaker, 2010).

### Sentiment Analysis and Cognition Engine

SEANCE has a user-friendly graphical user interface (GUI), where the user has to select the input folder that contains a file in.txt format and the output is saved in.csv format (Crossley et al., 2017). The software is freely available at https://www.linguisticanalysistools.org/seance.html. The SEANCE comprises preexisting words related to sentiments, cognition, and social order. These word vectors are taken from freely available dictionaries like SenticNet (Cambria et al., 2010a,b, 2012; also see Cambria et al., 2020 for recently released SenticNet 6), EmoLex (Mohammad and Turney, 2010, 2013), Lasswell dictionary lists (Lasswell and Namenwirth, 1969; Namenwirth and Weber, 1987), Harvard IV-4 dictionary lists used by the General Inquirer (GI; Stone et al., 1966), Geneva affect label coder (GALC; Scherer, 2005), and Affective Norms for English Words (ANEW; Bradley and Lang, 1999). It also includes Hu and Liu (2004) polarity indices for analysis of sentiments, mostly in social media contexts. Similarly, the Valence Aware Dictionary for Sentiment Reasoning (VADER) is more useful in classifying shorter articles related to social media text, movies, and newspaper articles (Hutto and Gilbert, 2014). Other than these databases, SEANCE also includes the Stanford POS Tagger (Stanford Core NLP; Manning et al., 2014) for verbs, nouns, and adjectives. It has the potential to report almost 3,000 indices, which may be a drawback sometimes, so 20 component scores were derived through principal components analysis (PCA) (Graesser et al., 2011; Crossley et al., 2015) to make it more manageable.

### Measures

#### Linguistic Inquiry and Word Count

The association between death, funerals, and emotions or affective processes (Gortner and Pennebaker, 2003; Glasgow et al., 2014) led to the selection of affective processes for further evaluation. Previous studies also identified words expressing emotions or affect significantly that influence linguistic dimensions (Wardecker et al., 2017; Khalil et al., 2018; Patro et al., 2018). The present research aimed to explore the association between linguistic dimensions such as function, words, and affect. The function words comprise articles, prepositions, personal pronouns, impersonal pronouns, auxiliary verbs, conjunctions, negation, and non-referential adverbs. In comparison, content words include nouns, verbs, adjectives, and common adverbs (Miller, 1995; Gamon, 2004; Jordan et al., 2019). The use of function words is also predicted to influence composite scores of analytical thinking and clout/confidence. The affective processes are related to positive emotions (love, nice, and sweet) and negative emotions (hurt, ugly, and nasty). It also incorporated anxiety- (worried and fearful), anger- (hate, kill, and annoyed), and sadness-related (crying, grief, and sad) words.

The second research aim was to identify the nature of the relationship between pronouns and social contexts. Funerals are practiced in different cultures according to their social norms and prevalent practices for ages. The frame of reference had always been social institutions and society, primarily including family and friends. Social processes included social-, family-, friends-, female-, and male-related words. Pronouns comprised personal pronoun (I, them, and her), first-person singular (I, me, and mine), first-person plural (we, us, and ours), second-person singular (you and your), third-person singular (he, she, her, and him), third-person plural (they and theirs), and impersonal pronouns (it, its, these, and those). Social processes are often studied using LIWC, as their relationships bind and impact various psychological processes (Golbeck et al., 2011; Boyd, 2017; Jiang and Brubaker, 2018; Li et al., 2020).

#### Sentiment Analysis and Cognition Engine

SEANCE helped investigate the link between “fear and disgust” and its association with adjectives, polarity nouns, and polarity verbs. The SEANCE results indicated that writers position emotions primarily in adjectives than in verbs followed by adverbs (Crossley et al., 2017). The fear, disgust, and negative adjective components included words from the EmoLex database, which had 3,324 negative emotions entries. SenticNet is the prime resource in the field of opinion mining and sentiment analysis. It has multiple versions; the current is SenticNet 6 and is a useful ML tool in polarity detection (Poria et al., 2013; Cambria et al., 2020). Polarity nouns and verbs mostly comprise words from SenticNet, which has a collection of around 200,000 words related to four affective dimensions, i.e., introspection, temper, attitude, and sensitivity (Susanto et al., 2020). These words are based on Plutchik's (2001) pioneering work on emotions and norms for polarity.

#### Bayesian Analysis

Owing to significant practical limitations of employing *p*-values for hypothesis testing (Sharpe, 2013), we used Bayes factors for hypothesis testing. The practice of null hypothesis statement testing started showing constraints in keeping pace with the knowledge advancement (Gigerenzer et al., 2004; Harlow et al., 2016). This led to a loss of confidence in empirical psychological researches (Ioannidis, 2005; Begley and Ellis, 2012; John et al., 2012; Nosek and Bar-Anan, 2012; Pashler and Wagenmakers, 2012; Button et al., 2013; Morey et al., 2016). Bayesian parameter estimation techniques have caught the attention of many researchers recently (Rouder et al., 2008; Lee, 2011; Lodewyckx et al., 2011; Wetzels et al., 2011; Kruschke et al., 2012). The Bayes factor hypothesis test is used to analyze the predictive efficacy of two statistical models on a continuum by quantifying the evidence for any change. The Bayes estimation incorporates prior knowledge (what was already known) and the likelihood (the extent to which the existing data will influence the previous data). The researcher can verify and estimate valuable information by selecting an appropriate prior distribution (Vanpaemel and Lee, 2012). It estimates and quantifies confidence that θ lies in a specific interval (Wagenmakers et al., 2018a,b).

Bayes compares two models: one is a null hypothesis that supports the absence of the effect (H0: ρ = 0), and other is an alternative hypothesis that claims the presence (H1: ρ = α). After full specification of the two competing hypotheses, Bayes probability rules are as follows:

$$\begin{array}{l} p(H1|\text{data})/p(H0|\text{data})=\{p(H1)/p(H0)\}*\\ \text{Posterior odds Prior odds}\\ \;\\ \;\{p(data|H1)/p(data|H0)\}\\ \text{ Bayes factor}\;{\text{BF}}_{\text{10}} \end{array}$$

The formula for the prior model indicates {*p*(H1)/*p*(H0)} as the relative probability of the prior odds before observing the data. After seeing the data, the posterior model is represented by {*p*(H1|data)/*p*(H0|data)}, that is, quantifying the relative plausibility of any change. The change from prior to posterior is estimated as the Bayes factor, BF_10_, and represented by {*p*(data|H1)/*p*(data|H0). Thus, both models provide a probabilistic prediction, and the model with the best prediction is accepted for further inference (Wagenmakers et al., 2018a,b). According to Raftery (1999), the Bayes factor provides a solution to hypothesis testing and model selection by acting as a thermometer to measure the intensity of evidence. The inferential end goal in the parameter estimation through Bayes analysis is the posterior distribution. JASP (jasp-stats.org; JSAP Team, 2016), a free and user-friendly statistical software with a GUI similar to that of SPSS, was used for both descriptive and Bayes analysis (Marsman and Wagenmakers, 2017; Love et al., 2019; van Doorn et al., 2020).

## Results and Discussion

### Function Words and Affective Processes

The first study question was to determine whether there is an association between the human affective process and the use of function words in newspaper articles published on funerals during the coronavirus pandemic. If so, then how strong is the relationship between the two variables? The selected 46 newspaper articles were analyzed to study using LIWC to quantify both syntactic features and psychological processes. Table 1 shows descriptive statistics and Bayes correlation analysis of both study variables. The mean score and SD are as follows: function words (Mean = 45.28, SD = 4.64) and affect/affective processes (Mean = 3.64, SD = 1.44). The skewness and kurtosis are well within the range for both affect and function words. Also, the *p*-value of Shapiro–Wilk test is indicative of a normal distribution.

**Table 1:** Descriptive statistics and Bayes correlation analyses of function words and affective processes extracted from newspaper articles on COVID-19 funerals using LIWC.

**Dimensions**	**Mean**	**SD**	**Skewness**	**Kurtosis**	***p*-value of Shapiro–Wilk Test**	**Pearson r**	**BF_**10**_**
Function words	45.28	4.64	−0.7	2.06	0.08	-	-
Affect	3.64	1.44	−0.2	−0.83	0.17	0.54	230

*COVID-19, coronavirus disease 2019; LIWC, Linguistic Inquiry and Word Count*.

**Figure 1:**
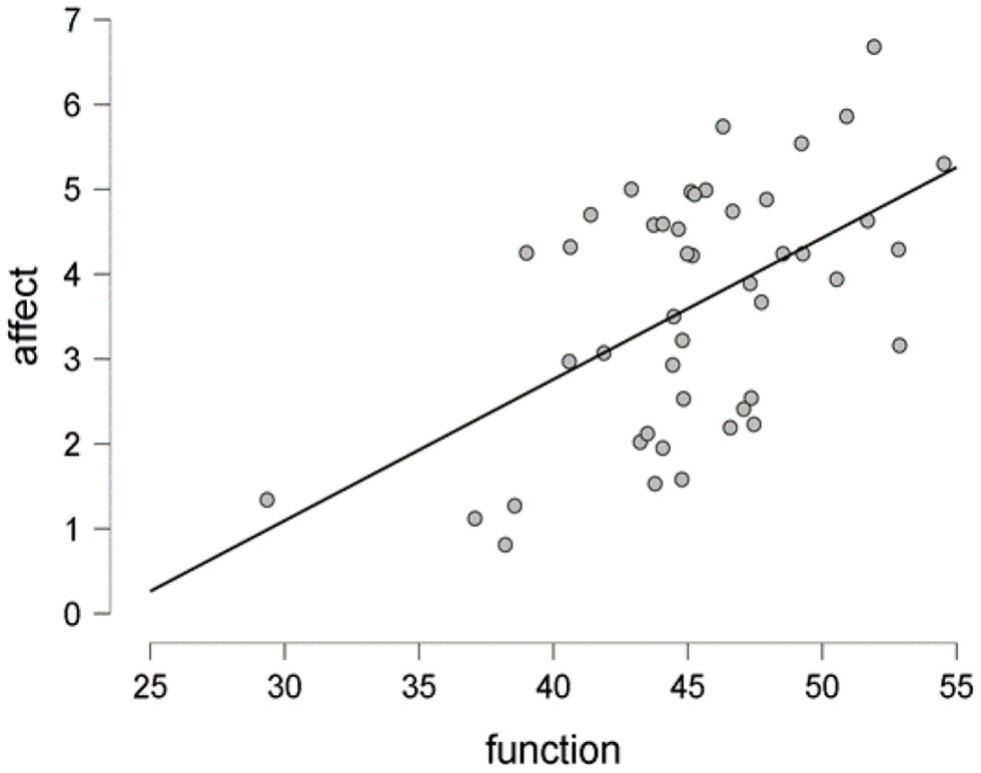
The scatter plot showing the proportion of function words vs. affective processes as per sentiment analysis of data collected from 46 newspaper articles on funerals during coronavirus disease 2019 (COVID-19) pandemic. The figure is based on JASP (0.12.2.0).

Now, we discuss the results of hypothesis testing. The null hypothesis states that there is no association between function words and affective processes (H0: ρ = 0). Particularly, we were concerned with the Pearson correlation ρ between the proportion of function words and affect. To explain further, we tried to examine the evidence that the data provided for the study hypothesis (H1). The first hypothesis (H1: ρ = α, α ≠ 0): newspaper articles will be more likely to reflect emotions using function words that express grammatical relationships with other words in a sentence. The scatter plot in Figure 1 shows a positive correlation (r = 0.54) with a 95% credible interval being in the range of [0.278, 0.704], which posits that there is a 95% probability that the correlation coefficient between function words and affect lies within the corresponding credible interval.

**Figure 2:**
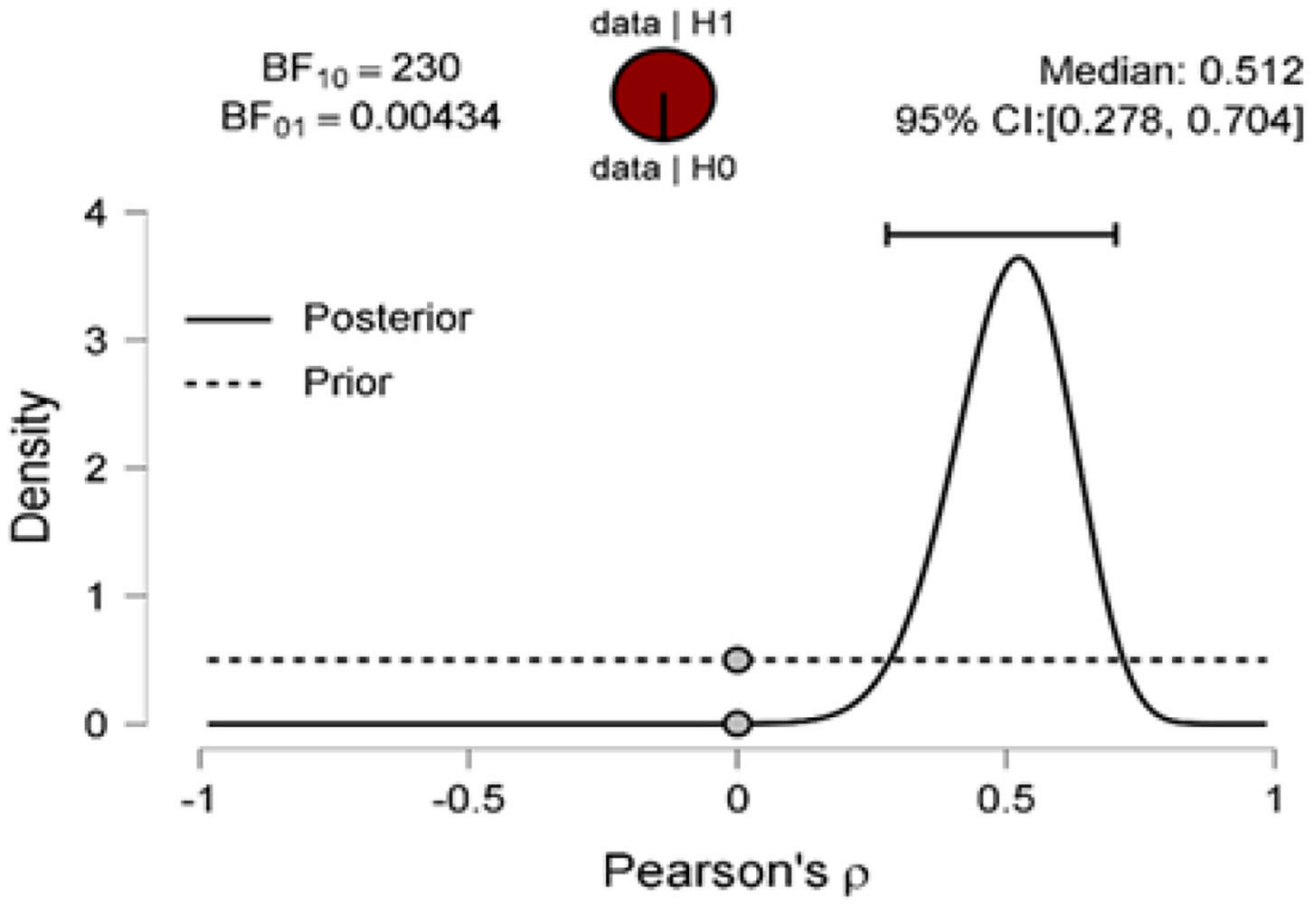
Prior and posterior distributions for the correlation between the proportion of the function words and affective processes as per sentiment analysis of data collected from 46 newspaper articles on funerals during COVID-19. The two-sided Bayes factor is visualized by the ratio between the prior and posterior ordinate at ρ = 0 and equals 230 in favor of the alternative hypothesis over the null hypothesis. The figure is based on JASP (0.12.2.0) (Wagenmakers et al., 2018a,b).

The Bayes factor is expressed as BF_10_ (and its inverse is BF_01_, i.e., 1/BF_10_) provides the intensity of the evidence that the observed data provide for H1 instead of H0. Here, Bayes factor was significant at BF_10_ = 230 (Figure 2), such that the observed data are 230 times more likely under H1 than H0. The proportion wheel gives a visual representation of the Bayes factor. Here, the corresponding proportion will be odds/odds + 1 = 230/231 = 0.996 (transforming to 0–1 interval); thus, the red area of the proportion wheel represents extremely strong evidence in favor of H1 covering more than 99% of the wheel. Figure 2 also presents the prior distribution for ρ under H1 (that is, the uniform distribution) and the posterior distribution for ρ under H1. The gray dots (visual representation of Savage–Dickey density ratio) showing the height of the prior and posterior distributions at ρ = 0 under H1. The ratio of these heights provides the Bayes factor for H1 vs. H0 (Wagenmakers et al., 2010).

The posterior mean of the regression coefficient of function words is 0.151 (Table 2). We can interpret it as one-unit increase in the use of function words intensifies affect by a gain of 0.151. The 95% credible interval is [0.073, 0.23], which signifies a 95% probability of regression coefficient of function words lying within the corresponding credible interval. The results (Table 3) show that BF_M_ = 181.444 shows extreme evidence for the model and that BF_10_ also supports the alternative hypothesis compared with the null hypothesis. In the model comparison Table 3, the P(M/data) column signifies the posterior model probability for both H1 and H0, P(M) is the prior model probability. As per the model comparison table, the probability of the model with the predictor (function words) has increased from 0.5 to 0.995. The *R*^2^ is the proportion of variance due to the predictor in the model. The *R*^2^ of 0.287 means that function words explain 28.7% of the variance in the psychological processes of affect. BF_inclusion_ indicates that there is extreme evidence favoring the alternative hypothesis compared with the null hypothesis (Ly et al., 2016). Our findings also find support in research done by Jordan and Pennebaker (2017) and Jordan et al. (2019), who were of the viewpoint that function words impact the attentional patterns and thinking style of people. Hawkins and Boyd (2017) also supported the use of LIWC language dimensions to provide valuable insight into human psychological processes.

**Table 2:** Posterior summaries of regression coefficients after accounting for the default priors for function words and the likelihood of the observed data.

**Coefficient**	**Mean**	**SD**	**P(incl)**	**P(incl/data)**	**BF_**inclusion**_**	**95% credible interval**	
						**Lower**	**Upper**
Intercept	3.64	0.182	1	1	1	3.294	4.018
Function	0.151	0.039	0.5	0.995	181.444	0.073	0.23

**Table 3:** Bayesian linear regression showing model comparison—affective processes.

**Models**	**P(M)**	**P(M|data)**	**BF_**M**_**	**BF_**10**_**	***R*^**2**^**
Function	0.5	0.995	181.444	1	0.287
Null model	0.5	0.005	0.006	0.006	0

Function words (Pozsonyi, 1938) have little lexical meaning or have an ambiguous meaning. Because of the functions they perform to express grammatical relationships among other words in a sentence or specify the attitude or mood of the speaker, their role in analyzing text cannot be overlooked. In our study, we found that the frequent use of function words in funeral-related newspaper articles have a greater likelihood of influencing emotional or affective processes.

### Pronouns and Social Relations

The second research question was to ascertain whether there exists any relationship between social processes and pronouns. If such association exists, to what extent does the data support the presence of a correlation? LIWC was used to analyze both pronouns and social processes in the newspaper articles written on burial rituals, rules, and regulations imposed by governments and public sentiments related to it. Table 4 presents descriptive statistics and Bayesian correlation analysis of both research variables. The mean score and SD are as follows: pronouns (Mean = 7.16, SD = 2.68) and social order (Mean = 10.63, SD = 3.24). The skewness and kurtosis factors are well within the range for both pronouns and social order. The *p*-value of the Shapiro–Wilk test is also indicative of a normal distribution.

**Table 4:** Descriptive statistics and Bayes correlation analyses of pronouns and social processes extracted from newspaper articles on COVID-19 funerals using LIWC.

**Dimensions**	**Mean**	**SD**	**Skewness**	**Kurtosis**	***p*-value of Shapiro–Wilk Test**	**Pearson r**	**BF_**10**_**
Pronouns	7.16	2.68	0.32	−0.02	0.9	-	-
Social processes	10.63	3.24	−0.06	0.64	0.14	0.65	19,748.55

*COVID-19, coronavirus disease 2019; LIWC, Linguistic Inquiry and Word Count*.

**Figure 3:**
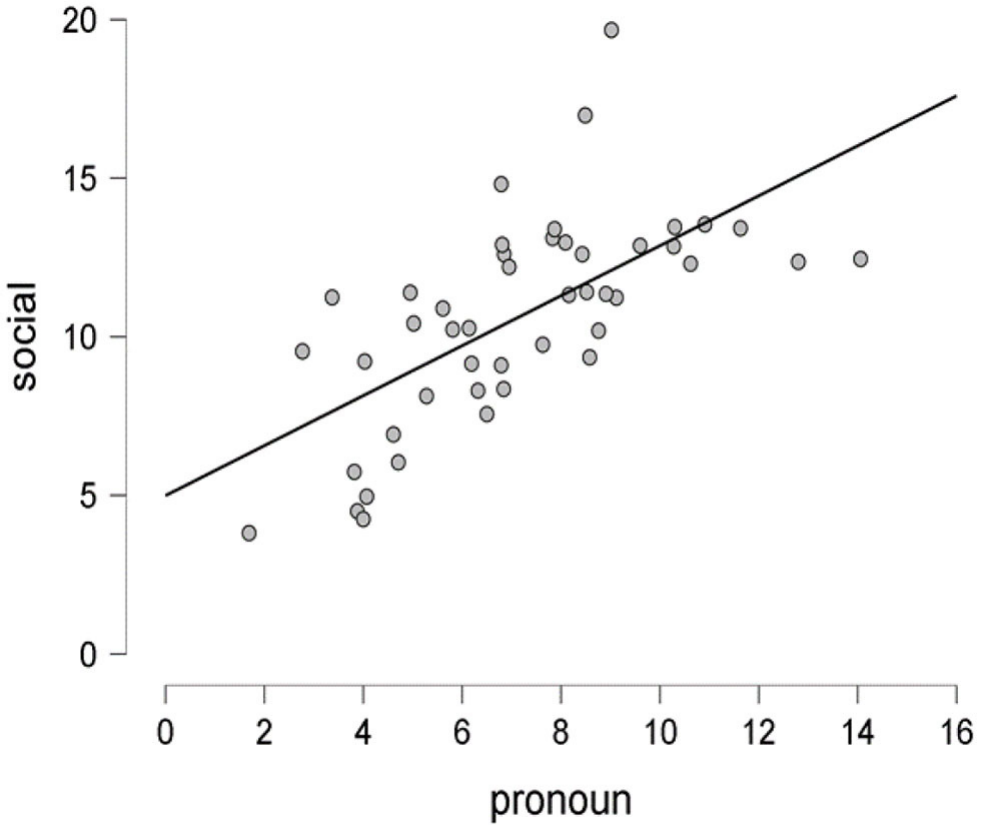
The scatter plot showing the proportion of the pronouns vs. social processes as per sentiment analysis of data collected from 46 newspaper articles on funerals during coronavirus disease 2019 (COVID-19). Figure based on JASP (0.12.2.0).

The null hypothesis states that there is no association between pronouns and social processes (H0: ρ = 0). The second hypothesis (H2: ρ = α, α ≠ 0) states that newspaper articles will be more likely to reflect social processes using pronouns that use words like I, me, you, they, we, his, her, and it to indicate family, friends, and other acquaintances in a society. The scatter plot in Figure 3 shows a positive correlation (*r* = 0.65) with a 95% credible interval being in the range of [0.427, 0.782], which posits that there is a 95% probability that the correlation coefficient between pronouns and social factors is within the corresponding credible interval.

**Figure 4:**
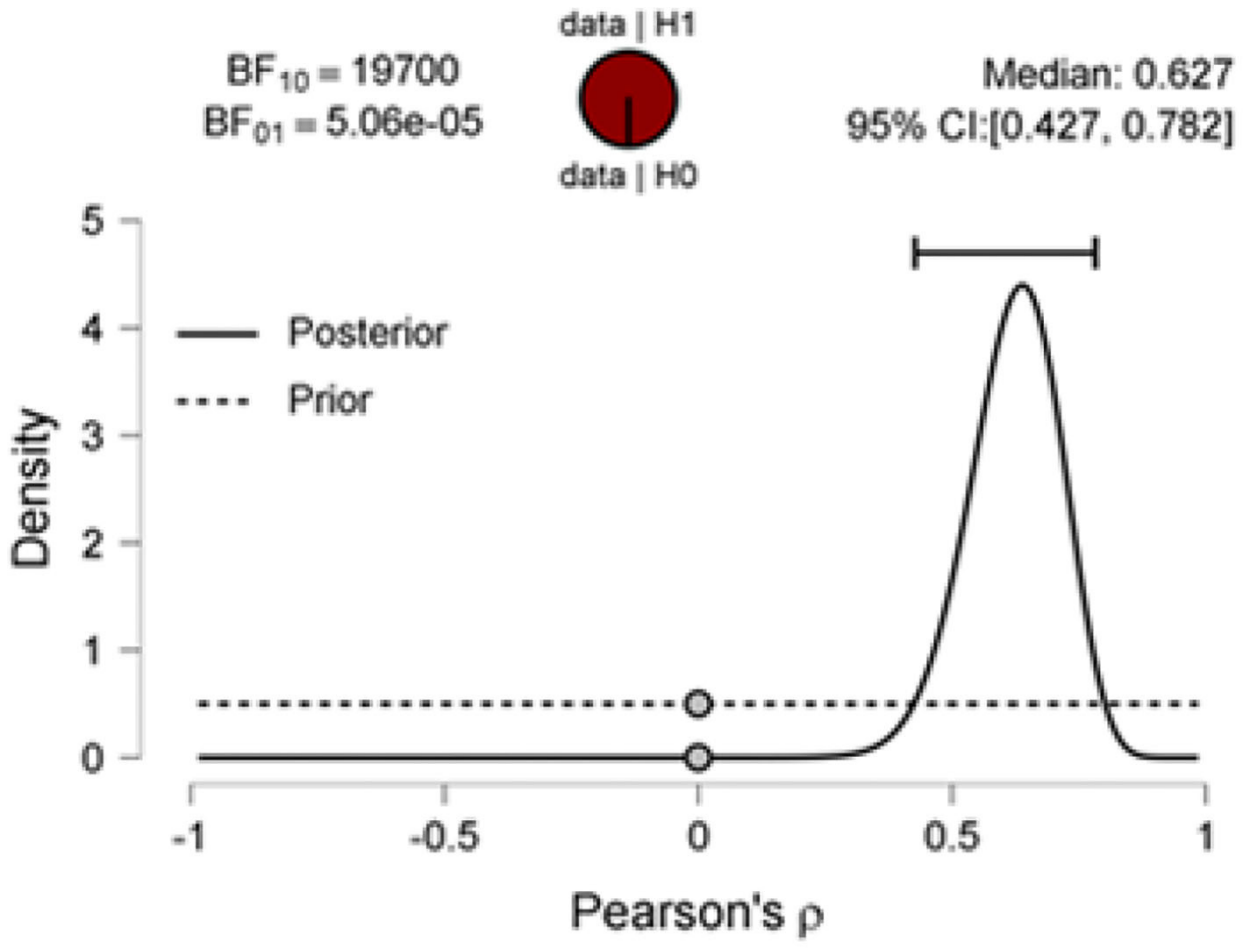
Prior and posterior distributions for the correlation between the proportion of the pronouns and social processes as per sentiment analysis of data collected from 46 newspaper articles on funerals during coronavirus disease 2019 (COVID-19). The two-sided Bayes factor is visualized by the ratio between the prior and posterior ordinate at ρ = 0 and equals 19,700 in favor of the alternative hypothesis over the null hypothesis. Figure based on JASP (0.12.2.0) (Wagenmakers et al., 2018a,b).

The Bayes factor was significant at BF_10_ = 19748.55 (Figure 4), such that the observed data are 19,748.55 times more likely under H1 than H0. The proportion wheel gives a visual representation of the Bayes factor. Here, the corresponding proportion will be odds/odds + 1 = 19,749/19,750 = 0.999 (transforming to 0–1 interval); thus, the red area of the proportion wheel represents extremely strong evidence favoring H2 covering nearly 100% of the wheel. Figure 4 also presents the prior distribution for ρ under H2 (that is, the uniform distribution) and the posterior distribution for ρ under H2. The gray dots (visual representation of Savage–Dickey density ratio) show the height of the prior and posterior distributions at ρ = 0 under H2.

Table 5 represents the posterior mean of the regression coefficient of pronouns. It can be interpreted as one-unit increase in pronoun impacting social components in the article by an increase of 0.742, nearly three fourth of an increase in the unit. The 95% credible interval is [0.497, 1.031], which signifies a 95% probability of the regression coefficient of pronouns lying within the corresponding credible interval. The results (Table 6) show that BF_M_ = 12,849.12 shows extreme evidence for the model, and BF_10_ also supports the alternative hypothesis compared with the null hypothesis As per the model comparison table, the probability of the model with the predictor (pronouns) has increased from 0.5 to 1.0. The *R*^2^ of 0.423 means that pronouns explain 42.3% of the variance in the psychological processes of social components. BF_inclusion_ (12,849.12) indicates that there is extreme evidence in favor of the alternative hypothesis in comparison with the null hypothesis. Kelter (2020), in research on null hypothesis significance testing in biomedical studies, draws a comparison between Bayesian inferential methods and conventional methods of analyzing *p*-values. Their findings suggest that the Bayes method using JASP provides an opportunity for the researchers to contrast both null hypothesis and alternative hypothesis before deciding in support or against any of the hypotheses. The social processes refer to social relations with family members and friends. Funerals indicate the loss and mourning of either a member of the family or friend. In funeral-related newspaper articles, frequent use of pronouns heightens our apprehensions about the health and well-being of close family members. We tend to be more alert when such relationship words are used that indicate the loss of someone forever. Further, the chance of mourning and other ritualistic ceremonies are not possible, which induces panic among the citizens in a country.

**Table 5:** Posterior summaries of regression coefficients after accounting for the default priors for pronouns and the likelihood of the observed data.

**Coefficient**	**Mean**	**SD**	**P(incl)**	**P(incl/data)**	**BF_**inclusion**_**	**95% credible interval**	
						**Lower**	**Upper**
Intercept	10.632	0.367	1	1	1	9.965	11.42
Pronoun	0.742	0.135	0.5	1	12,849.12	0.497	1.031

**Table 6:** Bayesian linear regression showing model comparison—social order.

**Models**	**P(M)**	**P(M|data)**	**BF_**M**_**	**BF_**10**_**	**R^**2**^**
Pronoun	0.5	1	12849.12	1	0.423
Null model	0.5	7.782e−5	7.783e−5	7.783e−5	0

### Polarity Nouns, Polarity Verbs, Negative Adjectives, Fear, and Disgust

The third research question was to explore whether there is any relationship between polarity nouns, polarity verbs, negative adjectives, and emotional components of fear and disgust. SEANCE, an ML tool, was used to analyze newspaper articles. The study hypothesizes (H3) that the word vectors related to polarity nouns, polarity verbs, and negative adjectives are strong predictors of negative emotions like fear and disgust. Table 7 shows descriptive statistics and Bayes correlation analysis of all study variables. The mean score and SD are as follows: polarity nouns (Mean = 0.22, SD = 0.16), polarity verbs (Mean = 0.31, SD = 0.17), negative adjectives (Mean = 0.81, SD = 0.65), and fear and disgust (Mean = 0.25, SD = 0.08). The skewness and kurtosis are well within the range for all the study variables. The *p*-value of the Shapiro–Wilk test also indicates a normal distribution for polarity nouns (0.4), negative adjectives (0.2), and fear and disgust (0.31) except polarity verbs (0.041). Even though they provide meaningful insights on the interrelationship of linguistic components with sentiments, cognition, and social order (Asghar et al., 2017; Crossley et al., 2017; Hamborg et al., 2019; Van Swol and Kane, 2019), because of their limited number (46 articles in total), generalizing the findings is not suggested.

**Table 7:** Descriptive statistics and Bayes correlation analyses of polarity nouns, polarity verbs, negative adjectives, fear, and disgust extracted from newspaper articles on COVID-19 funerals using SEANCE.

**Dimensions**	**Mean**	**SD**	**Skewness**	**Kurtosis**	***p*-value of Shapiro–Wilk Test**	**Pearson r (with fear and disgust)**	**BF_**10**_**
Negative adjectives	0.81	0.65	−0.46	0.15	0.2	0.49	64.903
Polarity nouns	0.22	0.16	0.28	−0.51	0.4	−0.56	431.147
Polarity verbs	0.31	0.17	0.57	−0.24	0.041	−0.57	685.947
Fear and disgust	0.25	0.08	0.13	−0.89	0.31	-	-

*COVID-19, coronavirus disease 2019; SEANCE, Sentiment Analysis and Cognition Engine*.

The third hypotheses were as follows: there is an association between negative adjectives, and fear and disgust (H3a: ρ = α, α ≠ 0); between polarity nouns, and fear and disgust (H3b: ρ = α, α ≠ 0); and between polarity verbs, and fear and disgust (H3c: ρ = α, α ≠ 0). Crossley et al. (2017) posits that negative emotions (like fear and disgust) are affected more by negative adjective and polarity verbs (negative) than by polarity nouns (negative). Our findings, however, show more support for polarity verbs (*r* = −0.57, BF_10_ = 685.947) and polarity nouns (*r* = −0.56, BF_10_ = 431.147) in contrast to negative adjectives (*r* = 0.49, BF_10_ = 64.903).

**Figure 5:**
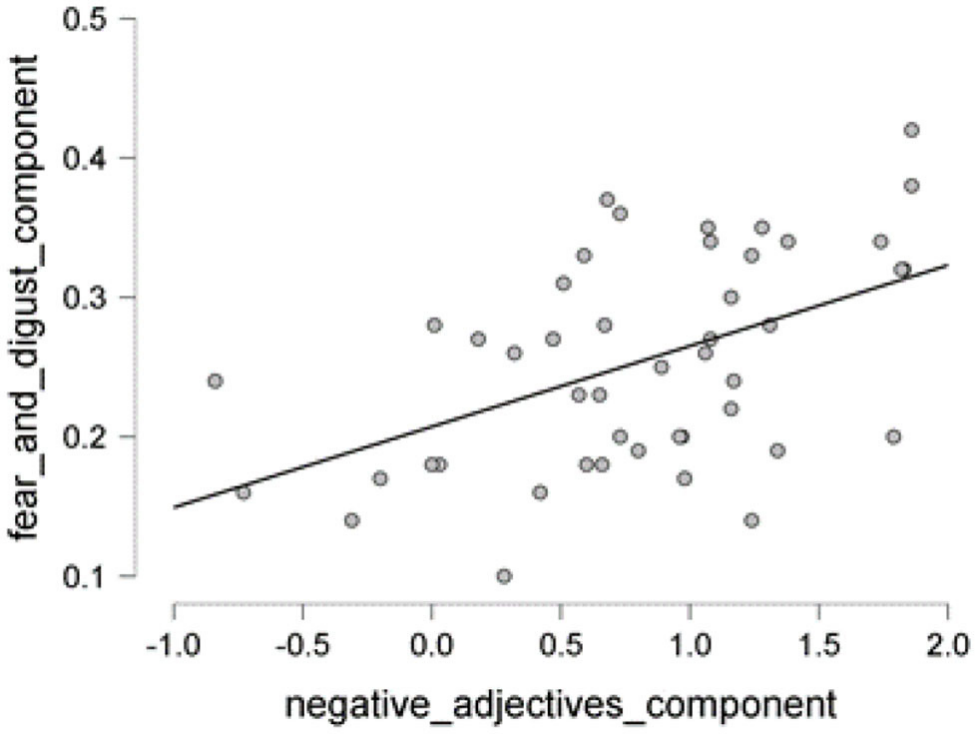
The scatter plot showing the proportion of the negative adjectives vs. fear and disgust.

**Figure 6:**
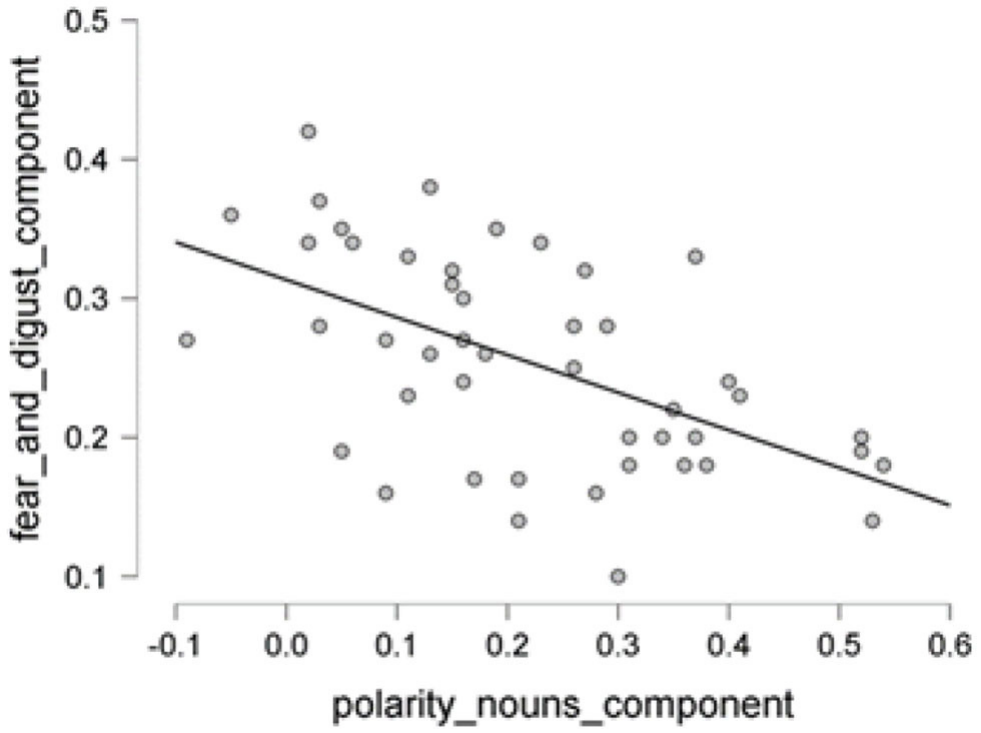
The scatter plot showing the proportion of the polarity nouns vs. emotional word vectors of fear and disgust.

**Figure 7:**
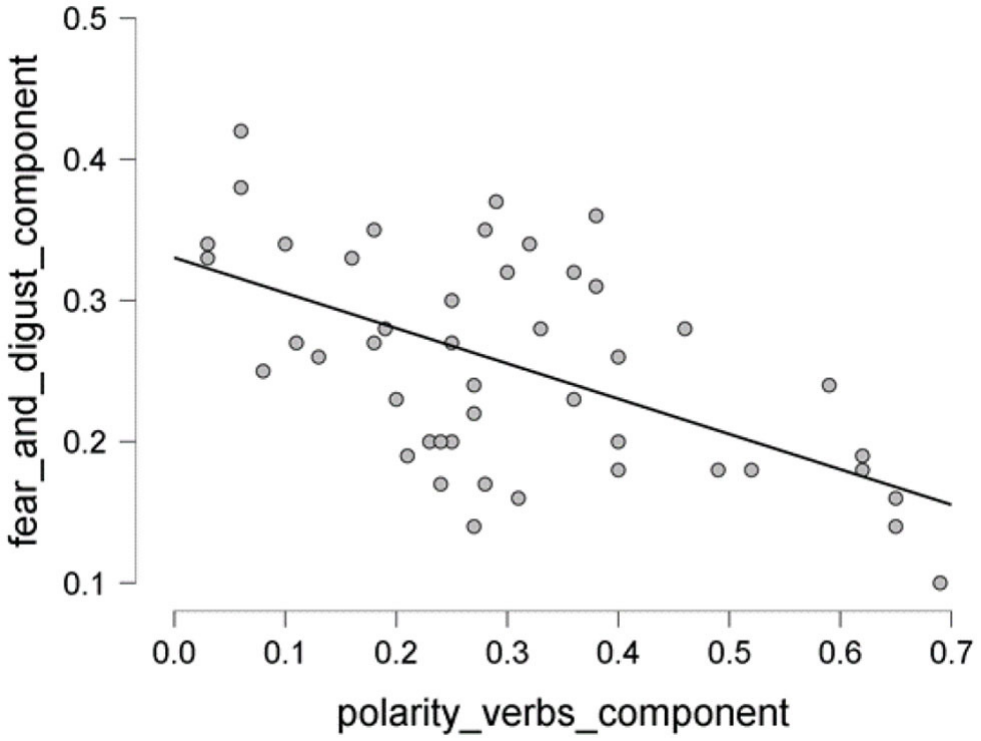
The scatter plot showing the proportion of the polarity verbs vs. components of fear and disgust.

**Figure 8:**
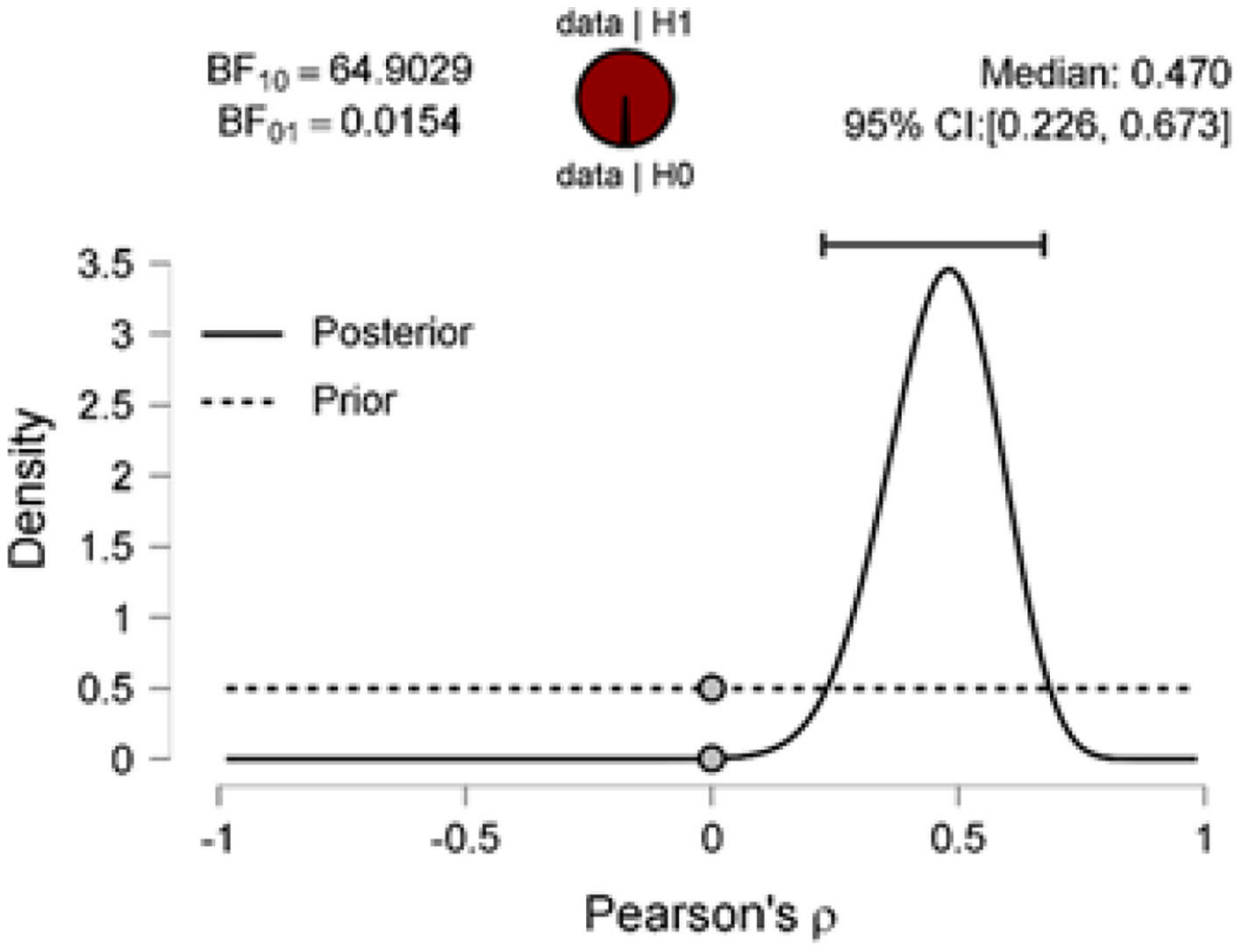
Prior and posterior distributions for the correlation between the proportion of the negative adjectives and fear and disgust. The two-sided Bayes factor equals 64.9029 in favor of the alternative hypothesis over the null hypothesis.

**Figure 9:**
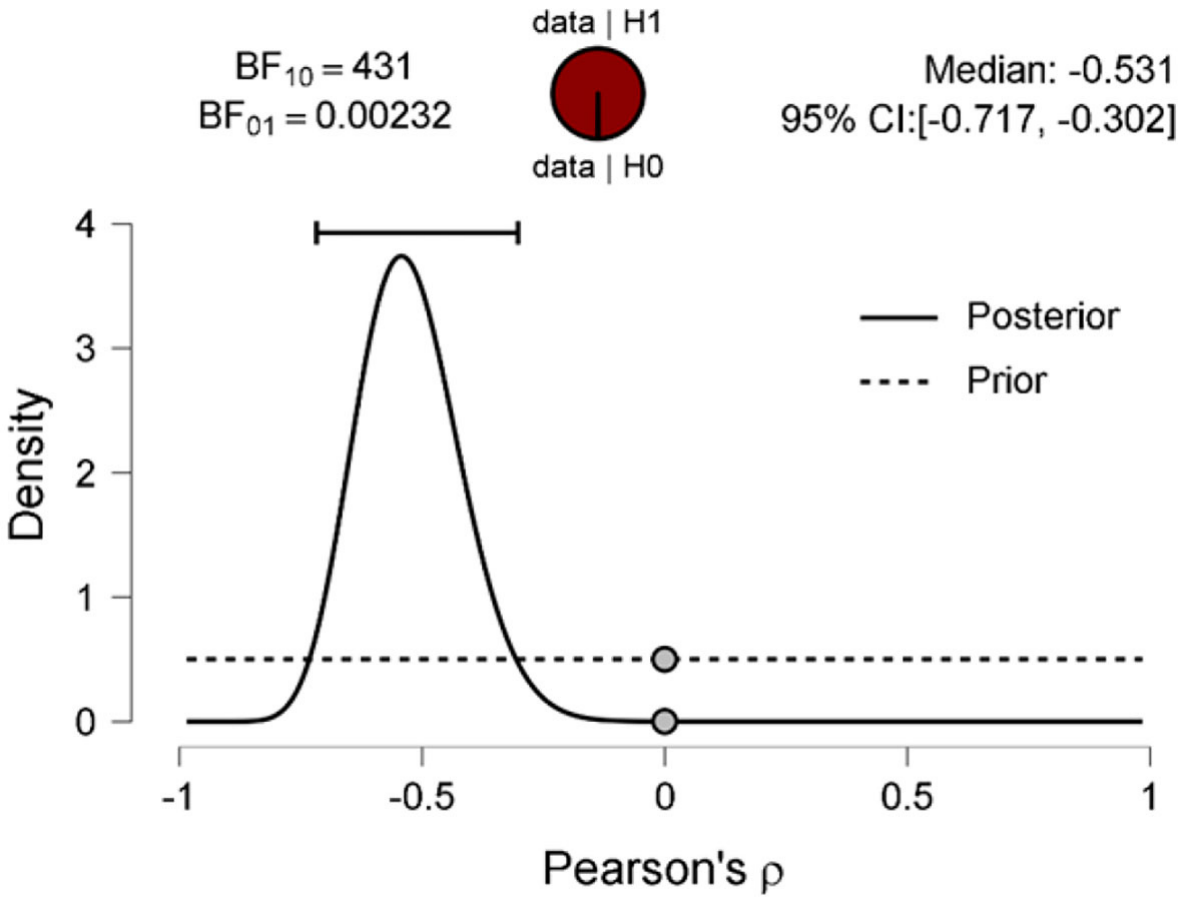
Prior and posterior distributions for the correlation between the proportion of the polarity nouns and fear and disgust. The two-sided Bayes factor is 431 in extreme favor of the alternative hypothesis over the null hypothesis.

**Figure 10:**
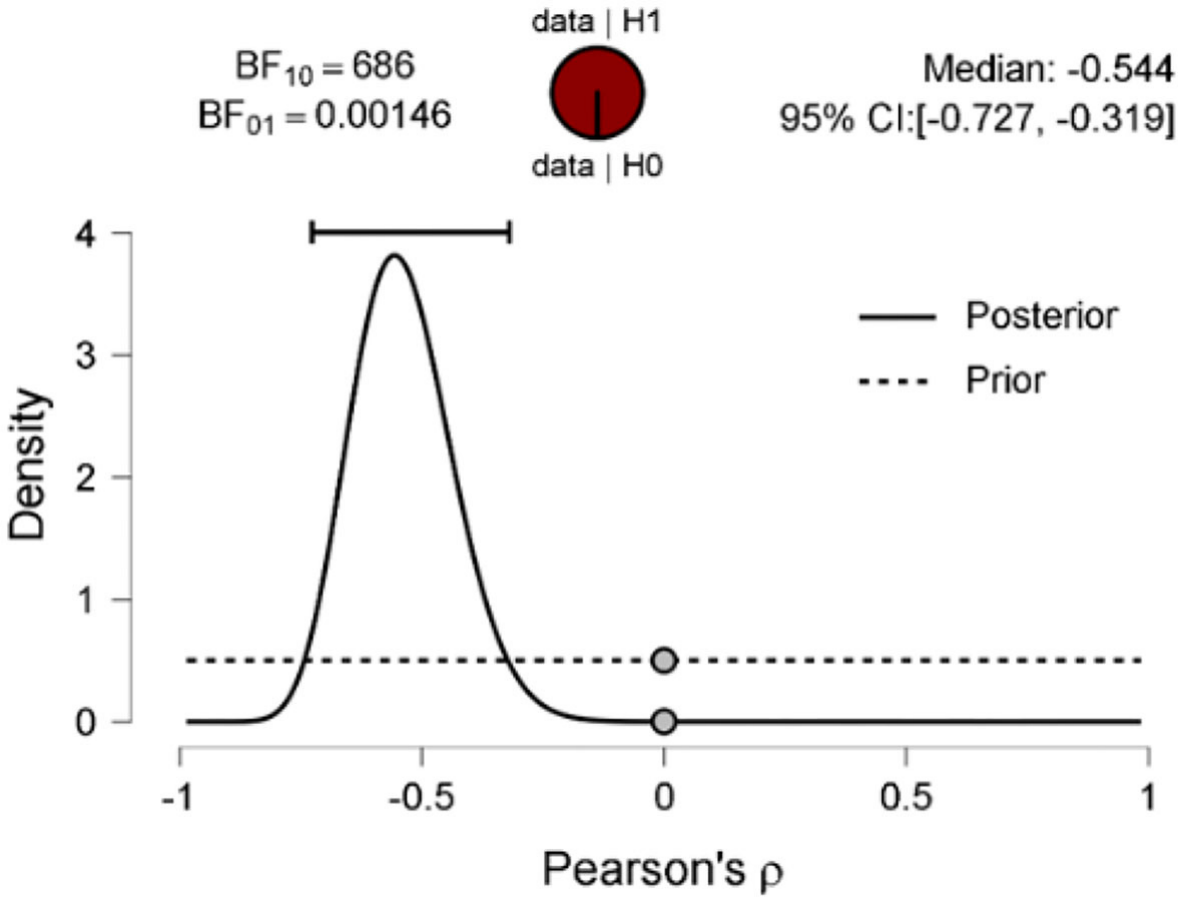
Prior and posterior distributions for the correlation between the proportion of the polarity verbs and fear and disgust. The two-sided Bayes equals 686 and presents extreme support for the alternative hypothesis over the null hypothesis.

The scatter plot shows a positive correlation between negative adjectives, and fear and disgust (Figure 5); a negative correlation between polarity nouns (Figure 6) and fear and disgust; and polarity verbs, and fear and disgust (Figure 7). The 95% credible intervals in Figures 8, 9, 10 show that there is 95% probability that the correlation coefficient between negative adjectives, and fear and disgust [0.226, 0.673]; polarity nouns, and fear and disgust [−0.717, −0.302]; polarity verbs, and fear and disgust [−0.727, −0.319] lie within the corresponding credible interval. Also, the proportion wheels in Figures 8, 9, 10 represent very strong evidence in favor of alternative hypotheses H3a (0.98), H3b (0.9976), and H3c (0.9985) in than the null hypothesis.

The posterior mean of the regression coefficient of negative adjectives is 0.025, polarity nouns are −0.195, and polarity verbs are −0.154 (Table 8). Overall, the results suggest that polarity noun relates to emotions or sentiments of fear and disgust in a negative manner more than polarity verbs and negative adjectives. The 95% credible interval for polarity nouns [−0.287, 0.089], polarity verbs [−0.263, −0.049], and negative adjectives [0, 0.053] also provide evidence in favor of the study hypotheses that certain language features are predictive of how writers use certain words to convey sentiments to readers. In Table 9, the best model that shows the maximum effect is the sum of all three components, BF_M_ = 19.618, which reveals strong evidence favoring alternative hypotheses. The *R*^2^ of 0.576 indicates a variance of 57.6% in the fear- and disgust-related words due to a combination of polarity nouns, polarity verbs, and negative adjectives rather than any single factor or sum of two factors.

**Table 8:** Posterior summaries of regression coefficients after accounting for the default priors for pronouns and the likelihood of the observed data.

**Coefficient**	**Mean**	**SD**	**P(incl)**	**P(incl/data)**	**BF_**inclusion**_**	**95% credible interval**	
						**Lower**	**Upper**
Intercept	0.254	0.008	1	1	1	0.24	0.27
Negative adjectives component	0.025	0.015	0.5	0.883	7.541	0	0.053
Polarity nouns component	−0.195	0.05	0.5	0.998	420.722	−0.287	−0.089
Polarity_verbs_component	−0.154	0.052	0.5	0.986	69.295	−0.263	−0.049

**Table 9:** Bayesian linear regression showing model comparison—fear and disgust component.

**Model**	**P(M)**	**P(M/data)**	**BF_**M**_**	**BF_**10**_**	***R*^**2**^**
negative_adjectives_component + polarity_nouns_component + polarity_verbs_component	0.25	0.867	19.618	1	0.576
polarity_nouns_component + polarity_verbs_component	0.083	0.116	1.445	0.402	0.52
negative_adjectives_component + polarity_nouns_component	0.083	0.014	0.154	0.048	0.465
negative_adjectives_component + polarity_verbs_component	0.083	0.002	0.019	0.006	0.404
polarity_verbs_component	0.083	5.904e−4	0.006	0.002	0.323
polarity_nouns_component	0.083	3.801e−4	0.004	0.001	0.308
negative_adjectives_component	0.083	6.424e−5	7.066e−4	2.222e−4	0.243
Null model	0.25	3.476e−6	1.043e−5	4.008e−6	0

In this section, verbs refer to the actions and/or behaviors toward others. Negative verbs used in the newspaper articles are indicative of a certain kind of contextually inappropriate behavior (by people other than the family) or action words (awkward, alienating/isolating, and didn't bow) related to death or loss of a closed one when there is no scope for mourning. Polarity verbs and nouns both are two-dimensional. While one dimension represents beliefs, actions, and things that are positive in nature, the other one is indicative of negative features. The abstract features of polarity noun use many words that are synonymous with pain, agony, frustration, and humiliation. Higher use of these words in funeral-related articles has the potential to incite fear and disgust among the readers.

The impact of written texts might have on the sentiments, and future behavior of individuals is an exciting field of study. The way news(paper) articles are written, the context, the meaning, and the inferences drawn need to be explored from an AI perspective given the volume of the text. Shaping the language to influence the audience is considered an essential skill. At the same time, it is important to gauge the sentiment of the public on how the same has impacted them positively or negatively. Similarly, Cambria (2016), in his paper on “affective computing and sentiment analysis,” raises concern about extraneous information confusing the global sentiments on the current problem. In the present study, we have attempted to examine the language used in the newspaper articles published on funerals during COVID-19 using LIWC and SEANCE. Our findings suggest that, first, frequent use of function words in funeral-related newspaper articles influences the emotional or affective processes; second, frequent use of pronouns or relationship words heightens apprehensions about the health and well-being of close family members; and third, higher use of polarity nouns in funeral-related articles incites fear and disgust among the readers.

## Conclusion

The indomitable truth of human life is death. All our behavior and emotions form a part of the journey from birth till death. Death is the culmination of a celebration called life. The undulated journey explains how humans experience their lives differently from each other. The fear of death, which the cessation of the unpredictable ebb and flow of day-to-day challenges, makes human life pleasurable for some and painful for others. As researchers from the field of Psychology, we are intrigued by death as a phenomenon, which binds everything mortal. People live in societies nurturing relationships, to get separated through unforeseen circumstances or foreseen and expected like death over the course of their lifetimes. We are bound and separated by cultures, religions, and customs. Nevertheless, funerals, a custom practiced by all communities, rituals, and traditions, are contrasting. The underlying sensibilities are complex and diverse; one may believe in life after death or rebirth, maybe, as many religions acknowledge reincarnations and faith in God.

For communities, traditional funerals positively channelize human fears and sorrows. The pandemic came as a great leveler with immense turbulence and emotional upheavals, followed by how humans deal with the unfathomable loss of family and friends. Fear of self-protection from an unknown virus changed everything that humans cherished on a social and community frontier. The real fear of an impending danger lingers outside our safe nests overwhelmed with love, warmth, and commitment for each other's safekeeping reflected through our behavior in dealing with the dead. The frequency and manipulation, though may be unintended, in use of language in articles on funerals like “Death had not fazed gravedigger …, a shiver runs up even his spine each time he sees a hearse pull up at the cemetery he tends,” published by India Today on May 18, have changed the course of rationality and sentiments of mankind all around the globe. The Guardian Australia published on April 13, commenting on police patrolling a funeral—“It was just disrespectful, to carry a gun in a Greek church, it's totally against our religion. But the way they came in, they didn't bow their heads or anything. They just started speaking to some of the people who were working in the church and taking notes as we're carrying out my dad.” An excerpt from a news article written in *USA Today* on April 2, 2020, stated: “Dying alone is the hardest part, but it's also really hard to grieve alone,”… “People think that doing a video conference or talking to the friends on Zoom or Zoom cocktail hour is awkward and alienating, but grieving alone is isolating.” A snippet of a news article posted by *Times of India* on April 10, 2020, stated guidelines on COVID funerals by the Government of India: “The crematorium/burial ground staff should be sensitized about Covid-19. The staff will practice standard precautions of hygiene, use of masks and gloves. Relatives may be allowed to see the face of the deceased by unzipping one end of the body bag (by the staff using standard precautions). Rituals such as reading from religious scripts, sprinkling holy water, and any other last rites that do not require touching the body can be allowed. The staff and families should perform hygiene after cremation/ burial.”

The conjoined anima and animus digging out of the obscured collective unconscious found its voice through the exaggerations of one-way channels of print and digital media. Here, psychology as a field of study of human cognition and the causality to infer from such a scenario lead us to the research. We wanted to explore how language is used to express emotions, care for society or close ones, and fear of losing them or life. Does the written expression of the media seem exaggerated to seek attention or make us more careful? Looking back, should we have acted in the manner we did? Does the news media influence our thinking style and corresponding action? There are many such tumultuous questions for which there are no answers as the virus still prevails. Have we adapted to the new normal?

## Limitations and Future Implications

Unlimited possibilities can be explored in the domain of psychological text analysis, about the availability of data and ML tools (technologically advanced open source software) that make analysis swift and simple. Language is the vehicle that psychologists employ to unravel the subliminal mysteries of humans, some of which are not known to them. The tools of AI, probabilistic processing of linguistic dimensions, psychological concepts, and cognitive neuroscience have advanced in combining technology and information across various disciplines. An automated analysis of subjective texts in print media and NLP in the electronic media makes working on big data a lucrative prospect, which was otherwise considered untenable in this work. These methods look promising, but it is easy to get tempted and fall for overgeneralization without verifying prior knowledge and adequate analytical skills. With extensive training and an innovative mind-set, the field of linguistic analysis using AI provides scope for cutting-edge research in social sciences.

## Data Availability Statement

The original contributions presented in the study are included in the article/supplementary material, further inquiries can be directed to the corresponding author.

## Author Contributions

SS and TS conceptualized the study and prepared the final draft for submission. SS collected and analyzed study data, wrote preliminary results, and wrote the preliminary manuscript draft. TS and RB provided critical feedback, reviewed the draft, and provided valuable inputs. All authors contributed to the article and approved the submitted version.

## Conflict of Interest

The authors declare that the research was conducted in the absence of any commercial or financial relationships that could be construed as a potential conflict of interest.
